# Effect of a Physical Exercise Intervention on Physical Function Parameters and Blood Analytical Changes in Lung Cancer Survivors: A Feasibility Study

**DOI:** 10.3390/clinpract14050173

**Published:** 2024-10-18

**Authors:** Teresa Soria-Comes, María Climent-Gregori, Inmaculada Maestu-Maiques, Ignacio Inchaurraga-Álvarez, Ferrán Cuenca-Martínez, Omar Cauli, Francisco M. Martínez-Arnau

**Affiliations:** 1 Medical Oncology Department, Hospital Universitario Doctor Peset, 46017 Valencia, Spain; soria_tercom@gva.es (T.S.-C.); maestu_inm@gva.es (I.M.-M.); 2 Pneumology Department, Hospital Universitario Doctor Peset, 46017 Valencia, Spain; climent_margre@gva.es (M.C.-G.); inchaurraga_ign@gva.es (I.I.-Á.); 3 Department of Physiotherapy, University of Valencia, 46010 Valencia, Spain; ferran.cuenca@uv.es (F.C.-M.); francisco.m.martinez@uv.es (F.M.M.-A.); 4 Department of Nursing, University of Valencia, 46010 Valencia, Spain; 5 Frailty Research Organized Group, University of Valencia, 46010 Valencia, Spain; 6 Chair of Healthy, Active and Participative Ageing, University of Valencia, 46010 Valencia, Spain

**Keywords:** lung cancer, rehabilitation, community-based programmes, quality of life, pre-albumin, muscular strength

## Abstract

**Background:** Lung cancer carries a high burden of systemic symptoms, including in survivors, leading to a reduced quality of life (QoL). We assessed whether a 12-week multicomponent supervised exercise programme, including muscular strength and aerobic training, was beneficial in patients who had undergone surgery for early non-small cell lung cancer (NSCLC) in terms of physical performance, QoL, and metabolic and nutritional analytical parameters. **Methods:** Physical performance was measured by gait speed, handgrip strength, 30 s sit-to-stand (30s-STS) test repetitions, distance covered in the 6 min walk test (6MWT), and the Short Physical Performance Battery (SPPB) score. QoL was assessed with the EORTC-QLQ-C30 questionnaire. Blood glucose, cholesterol, triglycerides, total proteins, albumin, pre-albumin, creatinine, c-reactive protein, insulin-growth factor 1 (IGF-1), and the haemoglobin and hematocrit percentages were measured before and after the intervention in order to observe any beneficial effects related to metabolic markers. **Results:** After the intervention, the mean scores for the 6MWT (*p* < 0.001), STS (*p* < 0.001), 6MWT (*p* < 0.01), and SPPB (*p* < 0.01) had significantly improved. However, handgrip strength and nutritional analytical were unchanged. The EORTC-QLQ-C30 functions and symptoms significantly improved after the intervention (*p* < 0.05 and *p* < 0.01, respectively). A significant decrease in cholesterol, triglycerides, and IGF-1 and a significant increase in pre-albumin in blood was also observed post-intervention (*p* < 0.05). **Conclusions:** This supervised, community-based 12-week multicomponent was feasible (adherence rate 70.35%) and provided benefits not only to physical performance but also to the quality of life of patients with NSCLC.

## 1. Introduction

Lung cancer is the second most common type of cancer and is the leading cause of cancer death in both men and women. Indeed, epidemiological data recorded in 2022 revealed that it was the most commonly diagnosed form of cancer worldwide (representing 12.4% of the total cases), the leading cause of cancer death (18.7% of all cancer deaths), and was responsible for 13% of new cancer cases [1]. Non-small cell lung cancer (NSCLC) accounts for 85% of all lung cancers [2], although survival is longer than in cases of small cell lung cancer. Lung cancer carries a high burden of symptoms, including dyspnoea, cough, fatigue, pain, depression, anxiety, and insomnia and thus results in a diminished health-related quality of life (QoL) [3,4]. These symptoms were reported by 35% of lung cancer survivors over 5 years, resulting in a lower health-related QoL [5,6].

One of the most common pathologies associated with lung cancer is chronic obstructive pulmonary disease (COPD) due to the strong involvement of smoking habits in the development of both illnesses. Furthermore, COPD severely limits the level of physical activity, especially in patients with recurrent exacerbations and further contributes to reduced QoL in lung cancer survivors [7,8]. Interestingly, among these patients, physical activity has been related to reduced fatigue, but many studies warn of inactivity and low exercise tolerance in individuals with COPD [8,9].

The most common treatment in the early stages of localised lung cancer is surgery, usually with adjuvant chemotherapy or chemoradiotherapy, which can induce, per se, a decrease in physical performance and QoL [10] (Phillips et al., 2022). Treatment for lung cancer impairs both functional and exercise capacity. For instance, six months after surgery, peak oxygen consumption in these cases is reduced by 13–28% [11]. In addition, the ability of these patients to transport and utilise oxygen and metabolic substrates during exercise is severely limited, contributing to exercise intolerance and fatigue. In this context, physical training has recently been introduced as a non-invasive intervention in patients with lung cancer, both preoperatively [12,13,14,15] and postoperatively [16,17].

The goal of physical training-based interventions is to increase exercise capacity in order to improve autonomy and health-related QoL, dyspnoea, and fatigue, as well as to alleviate possible psychological distress at all cancer stages [18,19,20]. Interestingly, studies in participants exercising pre-operatively reported improvements in exercise capacity but no change in health-related QoL immediately after the exercise intervention [16,21]. Other post-treatment (surgery, chemotherapy, or radiotherapy) exercise studies in patients with lung cancer demonstrated improvements in exercise capacity but produced conflicting results with respect to the impact of the intervention on health-related QoL immediately after the exercise intervention [16,22].

Exercise interventions, including home-based walking exercise training and weekly exercise counselling, were effective in improving the subjective and objective sleep quality of patients with lung cancer [23] as well as the symptoms of depression [18]. Moreover, exercise improved the functional outcome and symptoms for certain cancer populations, including those with lung cancer, when these programmes were hospital-based [24]. However, the feasibility, efficacy, and safety of structured exercise in patients with lung cancer is unknown. Community-based or briefer exercise interventions may be more feasible in this population, and the effects of and adherence to programmes should be evaluated before they are implemented at larger scales. Nonetheless, very few studies have examined rehabilitation programmes for community-dwelling individuals who had had lung cancer [25] or their impact on physical performance, QoL, or metabolic indexes.

Also of note, older populations with lung cancer that have undergone surgery are more susceptible to functional and cognitive deterioration after surgical treatment, which contributes to diminishing the real benefit obtained through this therapeutic approach [26]. Several studies have tried to assess the effects of physical exercise programmes on the recovery of functionality in patients who have undergone lung resection surgery secondary to a pulmonary neoplasm. However, to date, no supervised community-based intervention programmes have included these patients during the subacute post-surgical period in their active recovery programmes. Therefore, in this current study, we included patients with this profile, specifically those with cancer [27], in a multicomponent physical exercise programme based on global physical exercise guidelines (aerobic, strength, and balance [28,29]. Therefore, the main aim of this study was to assess the effects of a multicomponent supervised exercise programme, including strength and aerobic training, in patients who had undergone surgery for early non-small cell lung cancer on physical performance, quality of life, and metabolic and nutritional analytical (proteins and lipids) parameters.

## 2. Materials and Methods

### 2.1. Study Design

This project was a prospective, longitudinal, and within-group interventional study carried out specifically in patients attending the Medical Oncology and Pneumology Departments at the Hospital Universitario Doctor Peset in Valencia (Spain). The ethics committee at the same hospital approved the study on 26 October 2022 (protocol number 92.22), which also adhered to the Helsinki Declaration. This study was conducted with the informed consent of the participating patients.

#### 2.1.1. Participants

**Figure 1:**
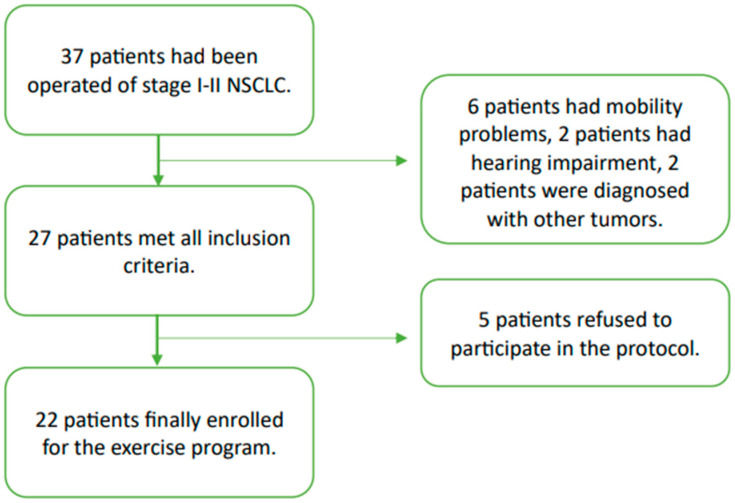
Flowchart to illustrate the participants’ recruitment process.

We included 22 patients (flowchart of enrolment in Figure 1) aged over 60 years with lung cancer who had undergone lung resection surgery during the previous 12 months for pathological stage I or II cancer according to the TNM classification (8th edition) and who had completed their adjuvant treatment if required (except where tyrosine kinase inhibitors [TKIs] had been indicated). In addition, all the participants had to be capable of independent ambulation (using technical aids if necessary but not help from another person) and had to give their signed informed consent to participate in the work. Any patients with a life expectancy of less than 6 months, who were institutionalised, with severe hearing or visual impairment, a contraindication for physical exercise (high cardiovascular risk factors), or with severe psychiatric diseases or moderate or severe cognitive impairments, were excluded. All patients in our department meeting these criteria were offered to participate in the study from the Pneumology or Oncology clinic. Recruitment was performed from October 2022 to March 2023.

#### 2.1.2. Intervention

The participants took part in a supervised, multi-component, community-based exercise programme at care centres for seniors owned by Valencia City Council. The programme was conducted in person, twice a week, over 3 months (between April and June 2023). Each session lasted approximately 60 min and comprised 3 phases as follows.

1. Warm-up and joint mobilisation (5–10 min).
2. Main part of the session (45–50 min): 3 blocks of 3 sets combining an upper limb and a lower limb exercise followed by a break of 120–180 s, during which aerobic (balance and/or cardiovascular) exercises were introduced.
3. Cool-down (3–5 min) with static stretching.

The exercise intensity was adjusted to the abilities of each participant, according to their individually perceived effort. The first 2 weeks were used for acclimatisation (an exercise intensity of 4 or 5 out of 10), followed by 3 weeks at a higher intensity (7 out of 10), then alternate weeks at maximum intensity (9 or 10 out of 10) and a high intensity (7 or 8 out of 10). To ensure the correct performance of each session and to adjust the exercises and loads according to the capacity and perceived exertion of each participant, the sessions were continuously supervised by a physiotherapist specialising in exercise with older people. In addition, the participants were encouraged to complete this exercise programme with 1 extra independent session of aerobic exercise (walking for 20–30 min) once a week.

#### 2.1.3. Data Collection

The study variables were collected at 2 time points: before the start of the intervention and immediately after its end. Each of the assessments comprised a series of clinical, functional and QoL variables and analytical markers. The clinical variables were collected from the Oncology Department medical records of the patients. Functional variables and QoL questionnaire data were collected directly by the investigators.

#### 2.1.4. Evaluation of Physical Performance

The functional variables assessed were gait speed, handgrip strength, 30 s sit-to-stand (30s-STS) test repetitions, and the distance covered in the 6 min walk test (6MWT). Gait speed was evaluated by tracking walking speed (best time from two trials) over a 4-m distance on a flat surface without the use of a gait-assistance device [30]. Handgrip strength was measured in the dominant hand using a 5030J1 Jamar Hydraulic Hand Dynamometer (Loughborough, UK). After sitting down, the patients were told to keep their arms hanging at their sides with their elbows bent. They were then instructed to hold the dynamometer as firmly as they could while maintaining straight elbow joints throughout the test, with a 60 s break between each attempt, recording a maximum of three attempts.

The 30s-STS test was also performed using a standardised protocol [31]. First, the clinician explained the test and ensured that the patient understood how to perform it. Then, the participants sat in a chair without armrests, positioned against the wall. Given that the 30s-STS test is thought to have strong test–retest reliability, it was only administered once [32]. The patients were told to perform as many standing cycles as they could in 30 s while keeping their arms crossed over their chests. Without using their hands, they were told to stand until they were completely upright and then to sit back down so that their buttocks contacted the chair. They received vocal encouragement during the test.

The 6MWT was performed in a straight 30-m corridor in accordance with guidelines for lung cancer patients [33]. Participants were instructed to walk as fast as possible for 6 min, and the total distance covered during that time was recorded. Their oxyhaemoglobin saturation was recorded throughout the test. Only standard verbal commands were given every minute in order to encourage the participants.

The short physical performance battery (SPPB) score was obtained after performing 3 assessments: standing balance, walking speed, and 5 s sit-to-stand repetitions (5s-STS). The standing balance test measures the ability of the participant to maintain three different foot positions—tandem, semi-tandem, and side-by-side—for a maximum of 10 s. Gait speed was measured in an obstacle-free 4-m corridor, allowing the use of gait-assistance devices if necessary. Finally, the 5s-STS task was measured as the time required to move from sitting to standing 5 times without using the upper limbs [34].

#### 2.1.5. Evaluation of Body-Mass Composition

Bioelectrical impedance analysis (BIA) was performed using a BF-300 instrument (Tanita, Tokyo, Japan) following a standardised technique to determine body composition [35]. While the participant was standing, 4 electrodes at a single frequency of 50 KHz and 550 mA were placed in a distal location (on the feet). BIA measurements were conducted early in the morning, ensuring that the patients (1) had not engaged in physical for a few hours; (2) had fasted for 2 to 3 h but had drunk plenty of water; (3) had urinated 30 min prior to the test; and (4) no metal objects were present during the test. After the patient was stabilised, the reactance and resistance values were noted.

#### 2.1.6. Evaluation of Health-Related Quality of Life

Health-related QoL was assessed using the European Organisation for Research and Treatment of Cancer Quality of Life Questionnaire (EORTC-QLQ-C30 scale, version 3), which has been translated into more than 40 languages, including Spanish [36]. Participants were evaluated based on 3 final scores: the global perception of health by the participants, functional status, and symptoms, each scored from 0 to 100. The scores on all the scales and single-item tests ranged from 0 to 100, with higher scores representing a higher response level. Thus, a high score for a functional scale represented better healthy functioning, a high score for the global health status represented a higher QoL and a high score for a symptom scale represented the presence of a high level of symptomatology. All the blood samples from which the analytical markers were extracted were obtained from the routine; conventional analyses carried out on the participants in the Hospital Universitario Doctor Peset laboratory (Valencia, Spain).

#### 2.1.7. Statistical Analysis

The SPSS software (version 27, IBM Corp., Armonk, NY, USA) was used for the statistical analyses. Descriptive data were presented as the median ± interquartile range (IQR) for continuous variables or as the frequency (*n*) and percentage (%) for categorical variables. The normality of the data distribution was assessed using Shapiro–Wilk tests. Paired Wilcoxon tests were performed to assess possible differences between the 2 time points. A 2-sided α probability of 0.05 was considered statistically significant. In order to account for multiple hypothesis testing, we applied the Benjamini–Hochberg procedure, which helped control the false discovery rate (FDR). By adjusting the *p*-values, this procedure helped us minimise the risk of falsely identifying significant associations. We considered any condition corresponding to an FDR less than 0.1 as statistically significant, ensuring that our findings were reliable. Effect size was calculated using r as the ratio of Wilcoxon’s z and the square root of the number of subjects. We considered r < 0.1 points as a very small effect, r = 0.1 points as a small effect, r = 0.3 points as a medium effect and r ≥ 0.5 as a large effect.

## 3. Results

Among 37 patients fulfilling the inclusion criteria of stage I-II NSCLC, 10 did not meet the inclusion criteria that prevented them from participating in the physical exercise program. The other five patients refused to participate because the days scheduled for the program were not suitable or because they said they did not like to do physical exercise. A total of 22 participants were included and accepted to participate in this study (*n* = 22, median age = 68 years, 31.8% female) (Figure 1).

All the participants had NSCLC, and most had been diagnosed with stage I NSCLC adenocarcinoma and had not received chemotherapy or radiotherapy. All the participants had been discharged after the surgical procedure, and a median of 5 months had elapsed after surgery. The clinical and functional characteristics of the participants at the beginning of the study are shown in Table 1. No modifications were made to the protocol during the study, and all the patients followed the programme described above, adapted to their functional status. No participant reported any adverse effects during the intervention. The adherence to the intervention calculated as a percentage over the total number of sessions was 70.35% (mean value) and 81% (median value).

After the intervention, we noted differences in functionality both in terms of gait speed (*p* < 0.001) and the 30s-STS test (*p* < 0.001). In addition, the distance covered by the participants in the 6MWT had increased significantly (*p* = 0.006), with clinically significant differences. However, there were no differences in handgrip strength. Furthermore, the SPPB score had increased, signifying that the risk of falls among the study participants had decreased and their functionality had increased. No differences were observed for the remaining comprehensive geriatric assessment variables or body composition parameters (Table 2).

The QoL scores reflected the improvement of the participants, with an enhancement in both functionality (*p* = 0.010) and symptom (*p* = 0.007) scores (Table 2). The improvement in the functional domain was the result of an improvement in all the subdomains: physical (*p* = 0.012), role (*p* = 0.042), emotional (*p* = 0.006), cognitive (*p* = 0.024), and social functioning (*p* = 0.049). The improvement in the symptoms was reported for fatigue (*p* = 0.006), pain (*p* = 0.018), insomnia (*p* = 0.032), appetite (*p* = 0.026), and constipation (*p* = 0.002). Adherence to the programme was high, with a median adherence over 80% (mean 70.35%). There was a positive correlation between adherence and a decrease in symptoms (rho = 0.549; *p* = 0.008). Similarly, correlations were observed between adherence and general health status (rho = 0.465; *p* = 0.029), physical function (rho = 0.424; *p* = 0. 049), role (rho = 0.649; *p* = 0.001), fatigue (rho = 0.670; *p* < 0.001), nausea and vomiting (rho = 0.497; *p* = 0.019), pain (rho = 0.637; *p* = 0.001) and insomnia (rho = 0.435; *p* = 0.043).

**Figure 2:**
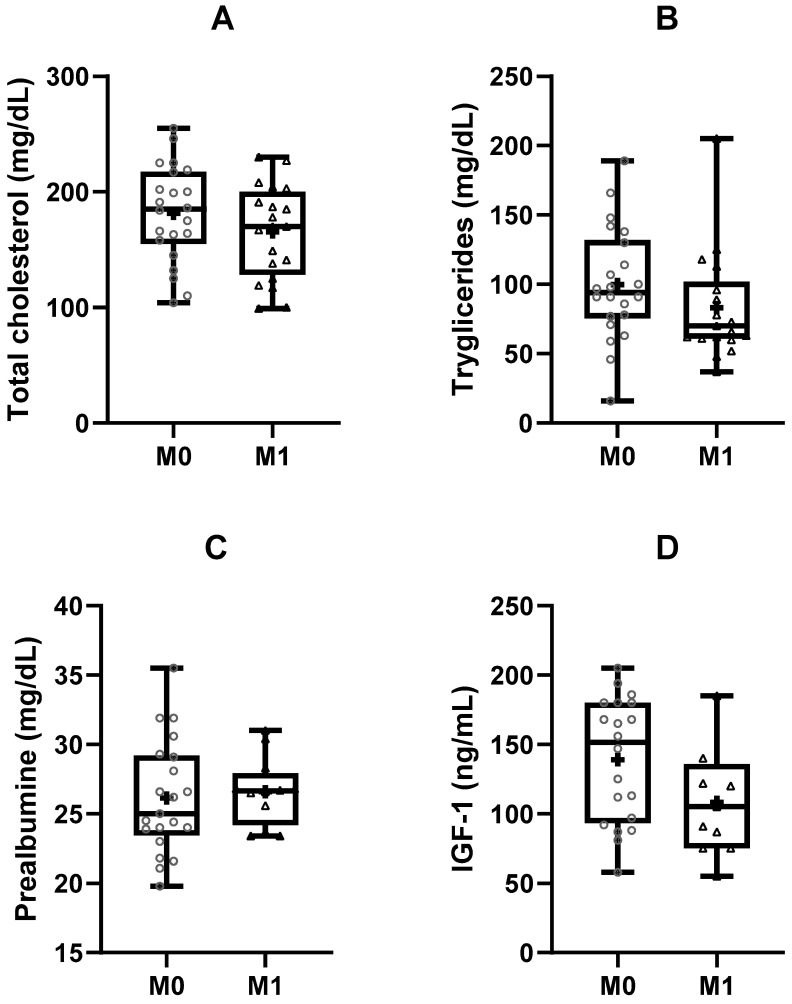
Metabolic changes detected after the exercise intervention. (**A**) total plasma cholesterol concentration; (**B**) plasma tryglicerides concentration; (**C**) plasma prealbumine concentration; (**D**) plasma insulin-growth factor-1 (IGF-1) concentration. M0: preintervention; M1: postintervention; *p* < 0.05 comparisons between the values measured pre- and post-intervention.

The positive results were accompanied by blood analytical changes in some parameters. These results were supported by a reduction in cholesterol and triglycerides in blood (*p* = 0.006 and *p* = 0.03, respectively), and an increase in pre-albumin (*p* = 0.034) and insulin-like growth factor (IGF-1) concentration (*p* = 0.024; Figure 2).

## 4. Discussion

In this within-subjects design study, we examined the effects of implementing a multi-component, community-based exercise programme, including strength, aerobic, and respiratory exercises, in patients with localised NSCLC aged over 60 years to help them in post-surgery rehabilitation. Most patients were male (68.3%), which correlated with the higher incidence of lung cancer in men worldwide, especially in Spain [37]. Although the majority of patients attempted the exercise programme, less than half were able to complete the intervention. Of note, the patients who completed the programme experienced an improvement in their lung cancer symptoms [24]. Adherence to physical activity interventions has been inconsistent in different studies, ranging from 45 to 85% in patients with lung cancer [38,39,40]. In our study, adherence was among the highest compared with previous studies, likely because the program was supervised [41,42,43]. The sample of lung cancer patients is generally “difficult” to engage in these interventions because most patients with lung cancer are insufficiently active or sedentary, and a series of studies reported a low adherence and high drop-out rate from physical exercise-based intervention in this population programs [44]. Among drop-out reasons, cancer-related side effects and, mostly, lack of interest and motivation represent key contributors. In addition, environmental and personal exercise preferences, fun, and social implications are important factors that influence the participation and consistency over time to a physical activity program [45]. In patients with lung cancer (and their caregivers), there is a higher risk of experiencing exacerbations of psychosocial distress because of the widely shared stigmatisation of this disease based on the close link between lung cancer and smoking [46].

In addition, the longer duration of our training protocol (12 weeks) could have influenced patient involvement. Furthermore, no agreements were made with patients regarding the definition of adherence or whether it includes attendance, session completion, or exercise intensity [47]. We defined adherence based on the attendance rate, and even though the completion rate in our study could be considered low, the benefits of the intervention were still clearly observed, as described below.

Our main objectives were to evaluate changes in parameters related to sarcopenia and QoL before and after the exercise programme. We found that both these factors were improved after the multicomponent intervention. In particular, a longer distance was covered in the 6MWT, and lower body strength was higher (according to the 30s-STS score) after the scheduled sessions. Several studies of pre/perioperative and postoperative training systems have shown improvements in the 6MWD outcome, as highlighted in multiple Cochrane reviews [11,12]. However, conflicting results have been reported regarding its relevance in predicting long-term postoperative outcomes [48]. Our study protocol included patients who had had thoracic surgery at least 4 weeks prior and showed that the programme had a beneficial effect on exercise capacity according to the 6MWD, even at this early stage. This was probably because of the longer duration of the training programme (12 weeks versus 4 to 8 weeks reported elsewhere). In support of these results, the gait speed (6MWD), 30s-STS score, and SPPB score (which includes balance, gait speed, and sit-to-stands) also improved.

Gait speed has been shown to be a prognostic factor clearly related to survival and mortality in older people [49], including individuals with haematological malignancies [50] or solid tumours [51]. This current evidence highlights the positive impact of integrating exercise programmes into the global management of patients with lung cancer—a group in which mortality remains worryingly high [52] despite advances in therapeutic pharmacological approaches, including directed therapies and immunotherapy. In fact, SPPB outcomes have been associated with the possibility of completing planned chemotherapy schedules and with the risk of adverse events in patients diagnosed with NSCLC [53]. Nonetheless, hand-grip strength, one of the main variables to define sarcopenia according to the European Working Group on Sarcopenia in Older People 2 (EWGSOP2; [54], had not changed at the end of our workout programme. In this sense, we hypothesise that the improvements in the outcomes of other tests involving walking or raising one’s own body mass were more important because they required musculoskeletal structures and respiratory, cardiac, and circulatory systems (all of which are affected by lung cancer surgery and can benefit from physical activity) to work sufficiently well in concert. In addition, the lack of significant improvement in the handgrip strength and lean body mass could probably be due to the type of intervention that was based on a global therapeutic exercise programme not being specifically designed to address the individual needs of these patients in specific parameters such as sarcopenia. Palmar grip strength requires specific exercises that focus on the muscles of the upper limb, and the absence of such a specific focus, as in the present study aimed at improving global functionality and quality of life, may result in failure to achieve significant results in this variable, as has occurred in previous studies [55]. On the other hand, it was not possible to analyse the results of the intervention on sarcopenia in the sample studied since none of the individuals included in the study met the criteria established by the EWGSOP2 [56], neither at the beginning nor at the end of the intervention.

Alongside prolonging survival, maintaining or improving QoL is one of the main goals of oncological treatments. Thus, one of the two primary objectives of this study was to evaluate QoL with the EORTC-QLQ30. There is substantial evidence to support the use of this scale in patients diagnosed with lung cancer [57], and it has also been used in other studies examining physical training [58]. Our results showed an improvement in functionality and a reduction in the symptomatic burden after completing the exercise programme, especially in terms of fatigue, pain, insomnia, appetite, and stress. However, other researchers only reported an improvement in dyspnoea but not in other symptoms [59]. Additionally, our patients reported higher scores in all the domains of the EORTC-QLQ30 (role, emotional, social, physical, and cognitive functioning), which highlights the strong relevance of including physical activity in the restoration of independence and well-being in patients diagnosed with early NSCLC.

A pilot study also reported an improvement in role functioning measured by the EORTC-QLQ30, but the results for the other domains and symptoms they evaluated were not statistically significant [60]. However, there were several key differences between the aforementioned pilot study and our study protocol: the exercise programme comprised an unsupervised walking regimen, did not include strength training, the participants had been diagnosed with advanced disease, and finally, compliance was lower than had been expected. Other studies were unable to demonstrate changes in QoL after a physical training programme [38,61], although some of these intervention programmes were shorter than our protocol, and others did not report the score of the specific components evaluated using this scale.

The time of initiation of the exercise programme after lung surgery has also varied between different studies, although early rehabilitation programmes (within 2 weeks post-surgery) improved respiratory functioning more than late intervention programmes (more than 14 weeks post-surgery), while other parameters such as physical functioning or QoL benefited equally from early or late programmes [48]. Nevertheless, the duration of the intervention is a decisive factor in determining their favourable effects [39,62]. In fact, in support of our results, interventions lasting 12 weeks or longer seemed to result in stronger improvements in QoL [40,59], and the latter intervention also improved anxiety and depression scores.

Surprisingly, body mass composition was not altered by the training intervention, as has been reported by other authors [40]. This could be the result of the nutritional intervention these authors implemented by adding the consumption of protein drinks/bars after each training session. Another difference that could explain the absence of changes in lean body mass in our cohort was the fact that our patients were free of disease after surgery, while those in the aforementioned study had been diagnosed with advanced cancer, also helping to explain why the median lean body mass was higher at baseline in our study (49.98 vs. 47.3).

Nevertheless, some analytical parameters had improved after our 12-week programme. Firstly, the lipidic profile was improved as a result of lower cholesterol and triglyceride levels, in accordance with previous evidence for chronic illnesses, including patients with breast or prostate cancer [2,63,64,65]. Second, levels of IGF-1 were also higher after the intervention. IGF-1 is a small polypeptide that mainly circulates bound to other proteins, which is involved in cell division, differentiation, metabolism, and apoptosis by mediating somatic growth and anabolic responses in different tissues. Furthermore, circulating IGF-1 can also influence carbohydrate and lipid metabolism in physiological and pathological conditions. The regulation of this molecule is considerably complex, although it has been established that protein intake is determinantal to IGF-1 levels and obesity is related to decreased levels of IGF-1 [66]. Thus, the latter could indirectly explain why we saw increased levels of IGF-1 in our study. Third, the median levels of pre-albumin were higher after our programme. Although these variables have been little studied in relation to physical exercise, pre-albumin seems to be a prognostic factor for survival in patients operated for NSCLC [67]. Finally, an analysis of patient survival was beyond the scope of this current work, but the improvement we saw in functional and analytical parameters suggests that supervised physical exercise programmes could have a positive impact on the main goals of oncological therapies.

To the best of our knowledge, this is one of the few studies that evaluated clinical, analytical, and QoL variables in patients who had undergone surgery for NSCLC after first completing a supervised, multicomponent training programme. Therefore, one of the most valuable strengths of this current research was our holistic evaluation of patients by taking a multidisciplinary approach. Unlike other unsupervised studies, our programme was conducted by a physiotherapist specialising in exercise in older people as well as by physical education professionals. With their guidance, the performance and capacity of the participants were maximised with a minimal risk of injuries.

Our main limitation was our relatively low sample size because only patients from one centre were included, as well as the absence of a control group. Thus, our findings must be confirmed in larger cohorts. These results should be interpreted with caution since the main limitation of our study is the small sample size with non-probabilistic sampling. However, in our opinion, the results obtained were sufficient for a pilot study and to create future research directions in this interesting field. Pilot studies are important in order to avoid significant errors before implementing large-scale studies such as this type of community-based intervention; they aim to assess feasibility and to obtain preliminary data that can be used to design a relevant, economical and statistically adequate large-scale study [68,69,70]. However, the fact that the design, content, and results from most pilot studies remain unpublished is, in our opinion, unfortunate for two reasons, e.g., the feasibility of the published data may prevent other researchers from making similar methodological mistakes and thus wasting scarce research resources. In addition, making pilot study results available may allow other researchers to avoid having to assess the feasibility of particular aspects of their proposed studies.

Physical activity and exercise are nonpharmacological interventions that have been shown to improve fatigue, quality of life, cardiorespiratory fitness, pulmonary function, muscle mass and strength, and psychological status in patients with lung cancer. Moreover, physical fitness levels, especially cardiorespiratory endurance and muscular strength, are demonstrated to be independent predictors of survival. Nevertheless, patients with lung cancer frequently present insufficient levels of physical activity and exercise, and these may contribute to low quality of life, reduction in functional capacity with skeletal muscle atrophy or weakness, and worsening of symptoms, particularly dyspnoea [44].

Furthermore, the proportion of male and female patients was not balanced in the sample by limiting the possibility of comparing the effects of the intervention in female versus male patients; this was due to the higher incidence of both NSCLC and smoking habits in men compared with women. Notwithstanding, positive effects were observed after the intervention, and this evidence could have implications for patients. Hence, we suggest that future decisions by multidisciplinary tumour boards include multicomponent exercise programmes in the therapeutic repertoire designed for patients diagnosed with early NSCLC run by physiotherapists and physical trainers. Moreover, our results raise the question of whether the improvements observed in cases of localised disease could also be achieved in patients with more advanced stages of cancer. Thus, future research must also include patients diagnosed with metastatic NSCLC [71] and should evaluate the impact of physical exercise both on clinical and analytical variables as well as progression-free and overall survival.

## 5. Conclusions

Results showed considerable benefits of a 12-week multicomponent supervised exercise programme in patients who had undergone surgery for early NSCLC in terms of physical functioning, QoL, and metabolic and nutritional analytical parameters. These results should encourage oncologists, pneumologists, thoracic surgeons and physiotherapists to work in cooperation to conduct clinical trials, including larger cohorts of patients with NSCLC to confirm the positive impact of these approaches. Therefore, in the near future, governments should invest in community-based programmes in order to implement supervised physical exercise in clinical practice among lung cancer survivors.

## Figures and Tables

**Table 1:** The characteristics of patients included in the study.

	Median (IQR) or Frequency (%)
**Age (years)**	68 (63.5–76)
**Sex (Female)**	7 (31.8)
**Civil status:**	
**Married**	15 (78.9)
**Divorced**	2 (10.5)
**Widowed**	2 (10.5)
**Coexistence:**	
**Living alone**	3 (15.8)
**Living with partner**	16 (84.2)
**Cancer stage:**	
**Stage I**	20 (90.9)
**Stage II**	2 (9.1)
**Time elapsed since surgery (months)**	5 (4–8)
**Chemotherapy (%)**	3 (15)
**Radiotherapy (%)**	3 (15)
**Presence of COPD**	15 (68.2)
**BMI (kg/m^2^)**	27.8 (26.3–31.5)
**Lean mass (kg)**	50.3 (43–59)
**Fat mass (%)**	31.9 (26.9–35.1)
**Ability to perform the activities of daily life (Barthel index score)**	100 (100–100)
**Nutritional status (MNA-SF score)**	12 (10.2–13)
**Physical functional status (SPPB score)**	11 (8–12)
**Comorbidities (CIRS-G score)**	7 (6–10)

Abbreviations: BMI, body mass index; CIRS-G, Cumulative Illness Rating Scale-Geriatric; COPD, chronic obstructive pulmonary disease; IQR: Interquartile range; MNA-SF, Mini-Nutritional Assessment Short-Form; SPPB, Short Physical Performance Battery.

**Table 2:** Changes in the study variables after the exercise intervention.

Variables	Preintervention-M0 (Median [IQR])	Postintervention-M1 (Median [IQR])	*p*-Value Adjusted	Size Effect (r) If Significant *p* Value (*p* < 0.05)
Gait speed (m/s)	1.09 (0.98–1.28)	1.61 (1.46–1.80)	<0.001	0.75
30s-STS (repetitions)	13.5 (10.8–14.0)	16.0 (15.0–19.0)	<0.001	0.77
Handgrip (kg)	30.0 (19.5–34.3)	25.0 (20.5–31.0)	N.S.	
6MWT (m)	436.0 (398.0–459.5)	471.0 (421.5–522.5)	0.012	0.58
Physical functional status (SPPB score)	11.0 (8.0–12.0)	12.0 (12.0–12.0)	0.007	0.66
BMI (kg/m^2^)	27.8 (26.3–31.5)	28.3 (25.6–31.3)	N.S.	
Lean mass (kg)	50.4 (43.0–59.0)	48.8 (43.4–58.9)	N.S.	
Fat mass (%)	31.9 (26.9–35.1)	31.2 (27.6–35.4)	N.S.	
EORTC-QLQ-C30 health-status self-perception (points)	52.0 (49.5–61.25)	51.0 (46.0–59.0)	N.S.	
EORTC-QLQ-C30 function (points)	23.5 (20.5–28.3)	22.0 (19.5–29.0)	0.016	0.55
EORTC-QLQ-C30 symptoms (points)	19.0 (16.0–24.5)	17.0 (14.0–21.0)	0.013	0.57
Glucose (mg/dL)	102.0 (95.0–114.5)	101.0 (94.0–113.5)	N.S.	
Total cholesterol (mg/dL)	185.0 (154.8–217.5)	170.0 (128.3–200.0)	0.013	0.58
Triglycerides (mg/dL)	94.0 (75.5–132.0)	70.0 (61.0–102.0)	0.033	0.46
Total proteins (g/dL)	7.3 (7.0–7.4)	7.1 (6.8–7.3)	N.S.	
Albumin (g/dL)	4.5 (4.3–4.6)	4.5 (4.4–4.8)	N.S.	
Pre-albumin (mg/dL)	25.0 (23.5–29.2)	26.7 (24.2–29.9)	0.037	0.45
Creatinine (mg/dL)	0.8 (0.7–0.9)	0.8 (0.7–0.9)	N.S.	
CRP (mg/L)	4.0 (2.0–7.0)	3.0 (1.3–4.0)	N.S.	
IGF-1 (ng/mL)	151.5 (93.3–180.0)	105.0 (75.0–136.0)	0.029	0.48
Hb (g/dL)	13.8 (12.7–14.9)	14.3 (13.2–15.2)	N.S.	
Haematocrit (%)	41.5 (38.9–44.3)	42.2 (39.8–44.3)	N.S.	
Haematites (×10^12^/L)	4.8 (4.4–5.0)	4.8 (4.5–5.0)	N.S.	
Leucocytes (×10^9^/L)	6.8 (5.6–8.1)	6.7 (5.4–7.3)	N.S.	
Platelets (×10^9^/L)	231.5 (198.5–260.3)	202.5 (181.8–229.5)	0.029	0.49

Abbreviations: 6MWT, 6 min walking test; 30s-STS test, 30 s sit-to-stand Test; CRP, C-reactive protein; EORTC-QLQ-C30, European Organisation for Research and Treatment of Cancer Quality of Life Questionnaire; Hb, haemoglobin; IGF-1, Insulin-Like Growth Factor 1; SPPB, Short Physical Performance Battery. N.S.: no significant difference (adjusted *p* > 0.05).

## Data Availability

The data presented in this study are available upon request from the corresponding author.
